# “You’re not just there to do a job”: a qualitative examination of Canadian long-term care worker strengths

**DOI:** 10.1186/s12913-026-14197-8

**Published:** 2026-02-25

**Authors:** Duneesha Goonetilleke, Nick Boettcher, Sofia Celis, Bonnie Lashewicz

**Affiliations:** Department of Community Health Sciences, Cumming School of Medicine, University of Calgary, 3330 Hospital Drive NW, Calgary, AB T2N 4N1 Canada

**Keywords:** Long-term care, Long-term care workforce, Person-centred care, Appreciative inquiry, Strengths-based approach, Qualitative secondary analysis, Thematic analysis, COVID-19, Canada

## Abstract

**Background:**

Research on the impacts of the COVID-19 pandemic on long-term care workers has largely emphasized the negative outcomes of crisis conditions and workforce distress, leaving strengths-based perspectives on workers’ experiences during this period underexplored. Recognizing the potential of strengths-focused inquiry to inform sector transformation and workforce renewal, we conducted a secondary qualitative analysis of Canadian long-term care worker experiences during the pandemic using an Appreciative Inquiry lens to examine and more fully represent workers’ strengths.

**Methods:**

A qualitative secondary analysis was conducted using an Appreciative Inquiry theoretical lens. Data were collected during the COVID-19 pandemic through semi-structured interviews with 50 long-term care staff members spanning diverse roles across 12 facilities in Alberta, British Columbia, and Ontario. Transcripts were analysed thematically in a process of moving from inductive familiarisation to deliberate application of an appreciative lens to identify successes, assets, and positive capacities within the long-term care workforce.

**Results:**

We present three themes identifying interlocking expressions of strength. First, during crisis, workers stretched roles, redistributed tasks, and supported one another to sustain care under extreme constraint. Second, staff upheld person-centred care in their daily actions, working from values of dignity, relationships, and residents’ goals. Third, workers translated experience into learning and advocacy, articulating ideas to improve staffing, role clarity, leadership practices, and visitation approaches. Across these expressions, workers’ tenacity, togetherness, and shared purpose enabled collective action, although these strengths often entailed costs such as fatigue and moral strain.

**Conclusion:**

Our findings add to existing literature on long-term care worker experiences during the pandemic by demonstrating that workers’ capacities for adaptability, person-centred care, and advocacy are vital assets for sector renewal. While amplifying and supporting worker strengths can enable meaningful transformation, these strengths alone cannot substitute for systemic investment and reform. To sustain care quality, workforce well-being, and crisis preparedness, interventions and policy design should embed worker perspectives to advance recognition and resourcing of their contributions. Deeper renewal also requires rewriting longstanding public narratives that undervalue LTC work.

**Supplementary Information:**

The online version contains supplementary material available at 10.1186/s12913-026-14197-8.

## Introduction

Although the World Health Organization declared an end to the COVID-19 pandemic on May 5, 2023 [1], its impacts continue to inform the lives of those who live and work in long-term care (LTC) homes and the futures of those who will in years to come. By LTC homes, we refer to residential facilities that provide 24-hour nursing and personal care for adults who require ongoing support over extended periods. These homes, variously termed nursing homes or residential care facilities across Canada and internationally, together constitute the institutional core of Canada’s LTC system [2]. As part of Canada’s rapid research response to severe workforce and mental health challenges during the pandemic [3], our research team observed how the demanding and often dire conditions of this period served as a litmus test of the depth and limits of workers’ capacities. Originally designed to examine worker mental health and pandemic preparedness, the qualitative interviews made evident the remarkable strengths of those who sustained the LTC system through the urgency of the crisis.

For this study, we revisited this latent topic within the pandemic worker interview data through an Appreciative Inquiry [4, 5] lens to examine and more fully represent workers’ strengths. We aim to promote deeper understanding, recognition, and organizational support for these strengths as a foundation for research, intervention design, and policy reform to cultivate workforce sustainability and readiness for future system demands.

## Literature review

The ways in which the COVID-19 pandemic exposed the cumulative effects of decades of austerity and systemic strain in Canada’s LTC sector have intensified attention to transformation for resilience, sustainability, and growth. To contextualize our secondary analysis of pandemic-era worker strengths, this review engages with three intersecting bodies of scholarship examining the sector’s structural conditions, the well-being of its workforce, and the need to rethink how knowledge about LTC is produced.

### Structural conditions of continuing care

Canada’s LTC sector is defined by the absence of unified federal regulation, a roughly equal mix of public and private ownership, and regulatory frameworks that differ across provinces and territories. These differences are expressed in funding decisions, staffing requirements, facility standards, and inspection practices [6]. While this plurality allows for local adaptation and experimentation, it has also yielded a sprawling, uneven policy landscape [7]. In contrast to Canada’s federally funded primary health care system, these structural conditions expose the LTC sector to market-driven pressures that prioritize cost containment and efficiency, with 29% of providers operating for-profit [8]. Critical scholars have long questioned this structural feature. To illustrate, Armstrong’s feminist political economy perspective emphasizes that “quality care” is inseparable from “quality work,” and argues that any system prioritizing cost containment over care labour conditions inevitably undermines both care outcomes and working conditions [9].

When COVID-19 struck Canadian LTC, the effects of chronic underinvestment, including lean staffing levels, contributed to devastating care outcomes system wide. More than 80% of Canada’s COVID-19 deaths in the first wave of the pandemic were among residents in LTC homes [10], and subsequent reports identified staffing failures as both a root cause of system fragility and a key driver of excess mortality [11]. International evidence has likewise drawn consistent associations of lower staffing levels and broader staffing instability with higher rates of COVID-19 infection, outbreaks, and mortality in aged care settings [12].

In the ensuing years, Canada’s national response has reflected a broader shift toward intergovernmental collaboration, though not to the extent of overhauling the constitutional autonomy of provinces and territories in governing LTC. An early federal intervention was the CA$1 billion Safe Long-Term Care Fund (2020), which provided short-term support for infection prevention and workforce stability [13]. Since 2023, ten-year bilateral “Aging with Dignity” agreements with every province and territory have allocated over CA$5.4 billion to infrastructure, infection-control standards, and workforce stabilization through wage top-ups, recruitment, and retention initiatives [14].

Beyond pandemic recovery, projected growth of Canada’s older population and the rising complexity of care needs are central drivers of investment in LTC. A national forecast estimated demand for continuing care will increase by 59.5% between 2019 and 2031, rising from 380,000 to over 600,000 Canadians [15]. Provincial projections further reflect this trend. In Alberta, demand for continuing care, including LTC, supportive living, and home care, is projected to rise by 80% between 2023 and 2033 [16]. In Ontario, a 2022 forecasting study estimated that by 2035 the province’s LTC sector will face a shortfall of nearly 28,000 full-time equivalent workers [17]. In unnerving contrast with the ambitious scale of workforce expansion required to meet projected demands, Canada faces an accelerating workforce shortfall in LTC. In 2023, a smaller share of the national health workforce was employed primarily in LTC than before the pandemic, with many shifting to jobs outside the sector [18]. In the nursing ranks, Canada lost approximately 2,500 LTC nurses between 2021 and 2022, a 5.1% decline in just one year [19]. In Ontario, pre-pandemic staffing data showed that half of all personal support workers (a role referred to as health care aides in Alberta) left the sector within five years, and 43% of those who exited cited burnout from working short-staffed as rationale for their departure [20]. This self-reinforcing structural challenge, in which workforce shortages trigger further departures, therefore predates but was amplified by the pandemic. Amid these conditions, the widening gulf between soaring demand and declining staffing points to everyday work lives that push workers out but, if better understood and valued, could also help retain them. To contextualize these dynamics, we now turn to scholarship on work-life experience and well-being in Canadian LTC.

### Lived experience and meaning in continuing care work

Working in LTC is demanding but can be deeply rewarding. The structural conditions detailed above are reflected in everyday pressures that workers endure and qualitative research from Canada vividly illustrated how pandemic conditions added layers of difficulty to these challenges. The pandemic caused profound social isolation, cumulative depletion, and a fading sense of work community [21]. These strains on well-being can manifest as burnout and moral distress. Moral distress is psychological suffering that arises when individuals recognize the morally right course of action but cannot pursue it due to external constraints [22]. A twelve-country scoping review of moral distress interventions in LTC characterized this widespread worker experience as a “second pandemic,” with few effective interventions [23]. Despite increasing acknowledgment of the scale and severity of these challenges, systemic responses remain limited and uneven across jurisdictions.

Even so, Canadian LTC workers ranging from care aides to nurses, allies, and managers have consistently reported notably high levels of positive job efficacy, in the sense that they find their work important and meaningful [24–26]. This efficacy is hypothesized to buffer concurrent burnout indicators such as cynicism and emotional exhaustion. More specifically, Health Care Aides have been found to score higher on professional efficacy than both nurses and managers [27]. Using related but distinct constructs, Rahman et al. found high levels of compassion satisfaction coexisting with moderate levels of burnout and compassion fatigue in an Alberta sample of 760 health care aides [28]. This suggests protective effects of the meaning that can be inherent in care work and this may be most pronounced among those who provide most of the direct care and face the greatest risk of burnout. Indeed, LTC workers themselves are well attuned to this contradiction surrounding meaning in care work. Czuba et al.’s qualitative study of work stress depicted caregiving as a “fluid experience” marked by continuous, dynamic tension between workers’ reasons for caregiving, and the burdens of unsustainable caregiving demands that often exceed formal job descriptions and available time [29, p. 4]. Overall, the ways in which sector features manifest in everyday realities of LTC work inform our analytic focus on worker strengths, which we now contextualize more closely in the final section of this literature review.

### Rethinking knowledge and strength in continuing care

In an important sense, Canada’s formidable LTC workforce challenges cannot be attributed to lack of knowledge. A major national pandemic-era briefing titled *Restoring Trust* examined the impact of COVID-19 in LTC and noted that 150 reports had already documented poor conditions in care homes across the country over the preceding decade [28]. The authors of this briefing reflected on how these many documents consistently detailed staffing shortages, under-regulated for-profit homes, chronic data deficits, and the gender-coded undervaluation of personal support work. Similarly, they found no shortage of recommendations to increase direct care hours, stabilize wages and benefits, collect comparable indicators, and enforce accountability. A tone of weariness is evident in the briefing’s observation that, despite the volume of documentation, “they all sit on a shelf. Nothing changes. Not really, not fundamentally” [30, p. 9]. A follow-up briefing identified 30 more reports published in the intervening three years and concluded bluntly, “the time for more reports is long past” [31, p. 5]. That the pandemic did not become the inflection point for reform that it initially seemed it could be is not lost on workers themselves. Longitudinal evidence from the Netherlands [32] showed that among healthcare providers, including those in LTC, an initial rise in perceived appreciation from employers and the public was already in decline by the second wave. This shift had measurable impacts on wellbeing, and qualitative analysis corroborated that early “applause and gifts” [32, p. 6] amounted to niceties that left the daily reality of work unchanged.

Given this, the LTC workforce can be read as inhabiting what Berlant calls the “crisis ordinary,” a state in which crisis conditions draw out into an unexceptional baseline atmosphere. Workers persist and adapt within systems caught in indefinite impasse, sustaining meaning while also in a state of “dithering,” “tottering,” and being “worn out by the promises they have attached to this world” [33, p. 28]. This same atmosphere that wears down workers also wears out the politics of knowledge production. As evidence accumulates to repeatedly confirm the same deficits, the goal of useful research may need to shift from producing more facts to the harder work of animating political and institutional attention.

Alongside health services research that continues to advance evidence on strength-based constructs such as LTC worker empowerment [34], meaning-of-work [35], and job efficacy [24–26], some contemporary LTC scholarship has sought to reorient evidence-making towards different ways of producing and mobilizing knowledge. One approach involves worker-driven co-production of research itself. Toms et al. [36] used the pandemic as an impetus to advance a recognition-based research agenda that positions care workers and residents not as data points, but as “experts by experience” capable of shaping both the questions and methods of inquiry. Recent participatory studies in Canada have demonstrated this broader approach using co-design and collaborative action methodologies to engage LTC workers in generating actionable solutions for workload stress, psychological safety, and mental health supports [21, 37]. Caveats to the epistemic potential of co-production include that it does not inherently escape familiar problem-solving deficit framings and that it requires time and resources often scarce in strained systems, which raises questions about who can realistically participate in transformative agendas.

A related direction in contemporary LTC research has centered the concept of joy. Notably, Braedley, Armstrong, and Klostermann [38] framed joy as a critical perspective for rethinking quality in LTC and, in so doing, challenged the dominance of efficiency, risk-based, and biomedical metrics of care quality. They position joy as a socially structured need and propose a taxonomy of belonging, meaning, sharing, and pleasure to redirect inquiry toward conditions that sustain its flourishing. By framing residents and workers as empowered co-creators of these conditions, joy is crafted as a social science concept demonstrating a politics of possibility oriented toward reanimating institutional attention. Modelling an applied example of this perspective, Hung et al. [39] examined joy not as a fleeting personal emotion but as a relational accomplishment embedded in structural context. Drawing on focus groups with twenty formal caregivers at a large public LTC home in British Columbia, the study explored how joy arises, is sustained, and how it materially supports retention and team resilience under pressure.

In all, the literature portrays LTC as suspended in a years-long pandemic aftermath, where concerns about the workforce and worker well-being are escalating yet the abundance of evidence has not translated into decisive change. At the same time, the enduring meaning of continuing care work remains evident, and current directions in scholarship such as recognition-focused inquiry, participatory co-design, and approaches that center joy present possibilities for renewal. Our analysis contributes to this broader turn in the scholarship as our purpose is to be part of systematically amplifying existing LTC worker strengths.

## Theoretical framework

We framed this secondary analysis with Appreciative Inquiry, a theory of change grounded in social constructionism and the generative power of inquiry to elevate human systems by focusing on their strengths, potential, and sustaining forces [4]. This theory treats inquiry as an intervention that co-creates reality within human systems by directing attention and opening future possibilities. Questions designed to elicit inspiration, hope, excitement, camaraderie, and joy activate natural inclinations toward energizing experiences that can be channeled into collectively desired organizational change [40]. In this way, Appreciative Inquiry produces knowledge that reflects a system at its highest values and aspirations and guides action toward preferred futures [41].

Among the numerous conceptual models in Appreciative Inquiry literature, we drew on Cooperrider and Srivastva’s (1987) four original guiding principles: (1) begin with appreciation; (2) be applicable; (3) be provocative; and (4) be collaborative. Consistent with the first principle, our research team’s shared appreciation for workers’ pandemic-era strengths, developed in our earlier analysis of worker mental health [3] prompted this secondary analysis and the decision to re-examine transcripts for strengths. The second principle emphasizes that knowledge should have practical application within the system studied. We therefore crafted findings using language and tone intended to ring true to the daily lives of participants and the wider community of LTC workers. Although especially visible during the pandemic, these strengths are not limited to that context and provide a foundation for future research and interventions to navigate post-pandemic realities and reimagine responses to enduring challenges in the LTC sector.

Provocation, the third principle of Appreciative Inquiry, calls for generating knowledge and images that provoke system members to see the open-ended evolutionary potential of their system and to inspire their capacities to engage in its evolution. Following pandemic-era Appreciative Inquiry scholarship that seeks what gives life meaning “even in the midst of the tragic” [42, p. 269], we acknowledge the validity of structural challenges in LTC, especially during the pandemic. Yet our adherence to provocation maintains analytic tension by foregrounding an image of worker strengths in counterpoint to deficit narratives of structural challenge. Finally, the collaborative principle holds that partnership between researchers and organisational members is essential to Appreciative Inquiry. Although our use of the theory is retrospective, the broader project was conceptualised with field partners, and participants were invited to steer interviews toward issues of importance. Additionally, two of the authors are/were themselves part of the LTC workforce, bringing lived experience of the system into the inquiry.

These four principles provided the overarching framework for our use of Appreciative Inquiry. To operationalize this approach in analysis, we also drew on the Three Phases of Strength Revolutions for Positive Organizational Development, namely Elevation, Configuration, and Refraction, which we adapted to guide theme development and sequencing [43]. Details of this adaptation appear in the analytic procedures described below.

## Methods

### Study context

This secondary analysis draws on interviews with LTC workers from three Canadian provinces, conducted within a broader mixed-methods project, “Supporting mental health and preventing moral injury among LTC workers”. Semi-structured interviews gathered accounts of working in LTC during the COVID-19 pandemic and its impacts on mental health and moral injury. The project was part of a Canadian rapid-response initiative to build pandemic preparedness in LTC, funded by the Canadian Institutes of Health Research and Healthcare Excellence Canada. Ethics approval was granted by the University of Calgary Conjoint Health Research Ethics Board, in accordance with Canada’s Tri-Council Policy Statement: Ethical Conduct for Research Involving Humans.

### Sample description and recruitment

This analysis includes 50 interviews with workers from 12 LTC organizations. Participants were recruited through partner organizations, professional networks, and word of mouth. Thirty were from Alberta, 19 from British Columbia, and one from Ontario. Workers represented a range of employment statuses and roles, including full-time, part-time, casual, and COVID-19–specific “pandemic hire” positions such as “screener” and “COVID-19 support lead.” Participants ranged in age from 19 to 71, with LTC experience from 3 months to 35 years, and educational backgrounds spanning high school to master’s degrees. Table 1 summarizes demographic details, including pseudonyms, education, job title, length of experience, and hours worked per week.

**Table 1:** Participant demographics (*n* = 50)

Role Category	Pseudonym	Job Title	Length of Time in LTC	Age
Leadership and Management	Cate	Clinical Nurse Leader	20 years	43
	Cynthia	Manager of Rehab Department, Occupational Therapist	20 + years	42
	Ella	Clinical Nurse Leader	18 years	46
	Erin	Director of Nursing	12 years	45
	Kayla	COVID-19 Support Leader / Day Program Manager	7 years	28
	Minsheng	Clinical Operations Supervisor	12 years	50
	Rhonda	General Manager	25 years	71
	Tyler	Director of Care	24 years	47
Direct Care	Adrian	Registered Nurse	21 years	49
	Amy	Comfort Care Aide	1 year	18
	Arianna	Health Care Aide	3 years	22
	Ava	Healthcare Support Worker	2 years	19
	Daisy	Health Care Aide	10 years	50
	Driya	Health Care Aide	1 year	19
	Emma	Comfort Care Aide	7 months	32
	Hanna	Comfort Care Aide	8 months	22
	Jasmine	Registered Nurse	9 years	51
	May	Health Care Aide	6 years	34
	Pascal	Licensed Practical Nurse	16 years	53
	Rebecka	Comfort Care Aide	1 year	20
	Rosita	Health Care Aide	6 years	NA
	Saana	Licensed Practical Nurse	6 years	46
	Shannon	Registered Nurse	1 year	22
	Valentina	Licensed Practical Nurse, Team Lead	11 years	32
	Violet	Licensed Practical Nurse, Team Lead	13 years	NA
Allied Health, Therapeutic and Psychosocial	Alexa	Recreation Therapy Assistant	6 years	29
	Alice	Social Worker	5 years	35
	Anna	Music Therapist	7 months	28
	Audrey	Activity Aide	1 year	22
	Braydon	Music Therapist	17 years	57
	Camila	Spiritual Health Practitioner	5 years	52
	Catherine	Dietitian	2 years	25
	Chantelle	Spiritual Health Practitioner	32 years	60
	Danika	Occupational Therapist	1 year	28
	Emilia	Social Worker	4 years	33
	Jenna	Therapy Aide	11 months	25
	Lily	Therapy Aide	9 years	38
	Lucy	Spiritual Health Practitioner	13 years	51
	Naomi	Social Worker	17 years	49
	Raina	Social Worker	3 years	45
	Raksha	Physiotherapist	23 years	47
	Sam	Social Worker	15 years	49
	Sarah	Recreation Therapist	6 years	31
	Sierra	Therapy Assistant	9 months	27
	Sophie	Occupational Therapy Assistant	14 years	35
Operational and Administrative Support	Clara	Screener / Receptionist	2.5 years	20
	Farbod	Dietary Aide	7 months	43
	Kathleen	Dietary Aide	1 year	33
	Lisa	Receptionist	3 years	59
	Tara	Human Resources Specialist	1 year	43

### Researcher positionality

The lead analyst, DG, is a young South Asian health care aide who began working in LTC in Alberta during the pandemic and continues to work with seniors in assisted living and complex dementia units. DG conducted the qualitative analysis in close consultation with co-authors. This insider perspective contributed sensitivity to worker strengths, while rigour was supported through reflexive practices including team memoing and analytic debriefs. SC conducted five interviews and contributed to interpretation. NB conducted five interviews and supported later rounds of analytic development and manuscript writing. The principal investigator (BL), whose practice background spans LTC, childcare, and disability services, also contributed to interpretation and writing. Our collective lived experience and motivation to improve the LTC sector were integral to the analysis, and our reflexive commitment kept interpretations grounded in the data.

### Data collection

Semi-structured, one-on-one interviews were conducted remotely via Zoom between March 8 and July 2, 2021, with participants joining from home or private workspaces. Interviews lasted approximately 55 min. An interview guide prompted discussion about sources of job satisfaction, stressors and experiences working through the COVID-19 pandemic (Additional file 1). Interviewers adapted their questions when they identified areas that required elaboration or when workers highlighted aspects that seemed particularly important or impactful to them. Audio was recorded and transcribed verbatim using Rev.com.

Oral consent was obtained before each interview. Participants were advised that discussing mental health could be distressing and were offered counselling referral information. They were reminded to share only what they felt comfortable disclosing. To preserve confidentiality, pseudonyms are used and potentially identifying details such as neighbourhoods, LTC facilities, and government ministers were not carried forward into the analysis or write-up.

### Data analysis

Analysis began during interviews as researchers acquainted themselves with participants. Insights from earlier interviews informed subsequent questions to evoke discussion on topics of importance to LTC workers. The research team met regularly to review preliminary findings and made early refinements to the interview guide. For example, following early accounts of extended overtime during outbreaks, we added the question, “What is the longest consecutive amount of time you’ve worked during the pandemic?”

For this secondary analysis, lead author DG led the analytic immersion and conducted the initial coding to capture patterned meaning. Thematic analysis then followed Braun & Clarke’s iterative steps, continuing through theme generation, review, definition, and refinement [44]. The analysis began inductively with repeated readings and detailed notes attending to both positive and negative experiences as well as articulated “needs,” given the broader project’s pandemic preparedness aims. This shifted to a more deductive interpretation as data were then coded with intentionally “appreciative eyes” [41, p. 97], focusing on worker strengths, successes, resilience, and attributes of the system’s positive core. The Master List of coded strengths captured workers’ commitment to care excellence and the assets they brought to LTC. This list was condensed into a Consolidated List of 28 codes to enhance cohesion and reduce repetition. Co-authors contributed to analyst triangulation in this consolidation process by reviewing the coded data against interview transcripts to resolve ambiguities and reach shared agreement on code definitions and inclusion through team discussion. Using mind maps, we clustered codes and generated candidate themes through iterative sorting and resorting. This produced a provisional thematic structure of three themes and seven subthemes, supported by a shortlist of illustrative quotes.

We returned to the Appreciative Inquiry literature as we collaboratively refined the structure and sequence of themes. This guided our decision to align the findings with the Three Phases of Strength Revolutions [5], which provided the scaffolding for our theme naming, ordering, and overall narrative arc. Elevation captured immediate demonstrations of strength during crisis; Configuration organized the motivational drivers that sustain person-centred care; Refraction highlighted learning, adaptation, and advocacy oriented to system change.

## Findings

We present three central themes that follow the progression of the Three Phases of Strength Revolutions for Positive Organizational Development [5]. The first highlights workers’ immediate demonstrations of strength during the pandemic, the second examines the deeper motivations that sustain their care, and the third raises possibilities for workforce and sector innovation.

### Theme 1: elevating strengths in response to a crisis - “We all rallied”

Workloads grew heavier during the pandemic. Residents who contracted the virus required more complex care. Safety and personal protective equipment (PPE) protocols made care safer but also more time-consuming. Restrictions on in-person visits introduced new demands for communication, emotional support, and alternative forms of family connection. These challenges were compounded by widespread staffing shortages. This theme profiles the strength of workers’ adaptability and dedication in response to these conditions, as evidenced in worker accounts of the expansion of formal roles, the strengthening of bonds with one another, and the sustained commitment to resident care.

#### Subtheme 1.1: stepping up to the challenge - “So I did everything”

Workers stepped up to fill shortfalls in direct care as roles that did not typically involve direct care were adapted beyond their usual scope. As a spiritual health practitioner, Chantelle’s ordinary role is providing emotional and religious support to residents and staff. Chantelle recalled assuming direct care responsibilities: “So, I did everything. I was feeding people, washing them, cleaning up their vomit”. She described once remaining at the bedside of a resident experiencing severe COVID-19 complications for fourteen hours during a shortage of health care aides. The resident had severe vomiting, and Chantelle held the bucket, cleaned, and changed them until they were transferred to the hospital. Chantelle provided this as one of many instances when she stepped up to assist nursing and care staff while also continuing, as much as possible, giving spiritual care to residents and helping staff navigate pandemic-related fear and distress.

Sierra, a rehabilitation therapy assistant, recounted similar scenarios of health care aides being “pretty spread out” which left her to step up, to the limits of her comfort and training, to assist residents with toileting, feeding, and portering. With less than a year of experience in LTC and not having been explicitly assigned such tasks, Sierra reflected on moments of hesitation where she asked herself, “wait, can I go help them? Is this appropriate for me to do?” Ultimately, she proceeded out of necessity, as “no one else was here to do it.” Danika, an occupational therapist, and Sam, a social worker, took on staff screening and COVID-19 swabbing. Sam summed up the overall situation among workers:

We’re in the middle of a crisis… So, it’s better to step up and provide where you can … than leave the place in a deficit. So, we all rallied, and we did what we could. (Sam)

This sense of teamwide mobilization patterned throughout workers’ accounts, with several using the phrase “all hands on deck.” For some, stepping into elevated work responsibilities was described as distinctly meaningful. Sarah, a recreation therapist, recalled putting her office tasks on the back burner to help on the unit, calling these “actually some of the most fulfilling days.” More broadly, the motives behind this shared resolve were often framed in practical terms, such as wanting to prevent the team from falling behind. Yet workers’ accounts also illustrated deeper layers of commitment through experiences of feeling seen, understood, and connected during shared challenges. Alexa, a recreation therapist assistant, described workplace relationships evolving within conditions of shared adversity and unfamiliarity, as workers began caring for one another on a “higher emotional level,” despite reduced physical proximity due to social distancing. Alexa went on to describe these strengthened bonds persisting after restrictions lifted, and others echoed the lasting impacts of this connectedness. When Ella, a clinical nurse leader, was asked what resources she used to stay well during the pandemic, she mentioned counselling through her employee and family assistance program but found it inadequate. Speaking remotely with a professional who lacked the lived experience of her work through outbreaks led her to “feel silly,” noting that despite her efforts to open up, “there was nothing there.” Ella also found it difficult to open up even to her spouse, explaining, “nobody else gets it, (it’s) so unique”. Contrastingly, she felt more connected to and understood by her colleagues who shared the same intense experiences:

People who went through it with me, all we had to do was barely bring it up or look at each other, and we would break down in tears. (Ella)

For some, the emotional bonds formed through shared adversity were described as carrying an element of “trauma bonding” (Danika). Yet Danika and others also clearly expressed genuine support and strength within these connections. Katrine, a dietary aide, captured this simply: “Like, if you’re sad, they help bring your mood up again”. Daisy, a health care aide, likened workplace connections to those of a family, noting she and her LTC friends, including those in different facilities, often connected by phone to cheer each other up and remind each other: “Tap yourself, you did a good job.” Heightened connections were uniquely salient for staff whose roles involved supporting colleagues’ emotional and mental wellbeing. Jasmine, a registered nurse, described intensifying her efforts during the pandemic to provide confidential, tailored support to coworkers who were struggling. Across roles, workplace connections offered a stabilizing influence that helped workers manage strain while maintaining their commitment to care. Camaraderie and mutual support within these relationships were strengths instrumental in upholding the extraordinary level of care residents depended on at the height of the pandemic.

#### Subtheme 1.2: extraordinary commitments to care - “They’re counting on us”

Aside from escalating workplace responsibilities, workers navigated pandemic-related challenges in their personal lives. Nevertheless, many participants’ commitment to colleagues and residents was relentless and motivated by a sense of duty as LTC workers. We elevate this extraordinary commitment while recognizing that the strengths demonstrated at times became inseparable from the personal costs and internal conflicts they entailed.

During an outbreak that claimed the lives of 21 residents, Ella recalled managing staffing shortages and supporting staff who were terrified to come into work. She stayed away from home for seven weeks to minimize transmission risk to her family. Witnessing the intensity of what Ella was going through and concerned about her contracting COVID-19, her family asked, “Why are you doing this to yourself?” Her family implored her to “Walk away, quit.” Ella refused, citing her duty as a nurse:

Knowing that the people here have no one else but us, you realize you’re choosing work over your family. And being okay with that because that’s what you do as a nurse. (Ella)

Rhonda, a general manager, similarly exemplified leadership commitments in crisis conditions. At age 71, she described delaying retirement out of duty when the pandemic began. Rhonda was undeterred by concerns from others that she deemed ageist, paraphrasing as: “Well, do you realize how old you are? Are you sure you should go in there?” To cover for missing staff during an outbreak, she worked 14-hour shifts for 17 consecutive days, including Christmas, returning home only to eat and sleep. She acknowledged the emotional toll of projecting strength while privately struggling:

As a leader, you had to never show vulnerability. You needed to be there… but I went home and cried quite a few times. It wasn’t guilt. It was just the extreme grief. It becomes overwhelming. (Rhonda)

Rhonda’s leadership was not confined to administrative decision-making. She worked alongside frontline staff, and navigated delicate, morally laden decisions around resident isolation and end-of-life care. This experience gave Rhonda a “better appreciation for frontliners,” and, reflecting on the exhaustion and emotional demands this work entailed, she remarked that “we don’t give them enough accolades.” Cate, a clinical nurse leader, chose to stay with her team during an outbreak despite her mother’s terminal illness. With no additional resources to support the mounting workload, she anticipated that taking time off would leave a colleague alone to shoulder the responsibilities of two people. She recounted taking a single vacation day during the first year of the pandemic, which she used to fulfil her mother’s wish of going to the beach in the summertime. Cate reflected on her decision in terms of guilt attached to both options that she could not fully resolve:

I felt guilty that… maybe I should have stayed with her. But at the same time… suddenly the outbreak hit, and everyone was sick… So, part of me is guilty for… if I take the time off, there will only be one supervisor. (Cate)

Alexa described enduring a private dilemma during an outbreak. Feeling unwell from early pregnancy, she continued working without disclosing the reason to coworkers and grew anxious that colleagues might question her health or assume she had COVID-19 symptoms. When she later took time off due to a back injury, Alexa’s colleagues, now aware of her pregnancy, urged her to prioritize her health and that of her baby. Yet she experienced renewed internal tension between self-care and her sense of commitment to the team: “I want nothing more than to go back to work,” she said, adding that she hated to think of her “co-workers picking up my slack and stuff.” Other workers also illustrated commitment in terms of bearing high levels of physical strain. Danika, for instance, noted that although “all of us had shoulder pain and back pain at some point,” workers felt “morally obligated” to keep going.

Daisy’s account puts a fine point on the physicality of this moral commitment. She illustrated what it took to get through shifts in dangerously short-staffed conditions, with only three workers covering a floor when even six or seven would have been barely sufficient. She described skipping breaks, forgoing meals, frantically donning and doffing PPE, and literally running between residents, clarifying: “It’s not brisk walk, we are really running.” She spoke candidly about the toll these conditions took in terms of reaching the physical limits of commitment:

It’s just heartbreaking, because you’re trying to be strong, yes, but it’s your body that really can’t cope up anymore. Your mind is so into it. (Daisy)

Building on this portrayal of mental and emotional commitment, Daisy described intensified connections with residents during outbreaks and the internalized responsibility of knowing that residents depended solely on workers:

We love them more. We don’t mind if they are [Covid] positive or not. You love each other more, even though it’s hard… as long as I’m taking care of everything, like really following the protocol, I’m not afraid, because they’re counting on us. That’s what I know. They’re counting on us. (Daisy)

Through our Appreciative Inquiry lens, the worker strengths elevated in this theme, particularly the exhaustion and pain displayed amidst extraordinary commitments to care, provoke careful interpretation of their meaning and implications including to avoid romanticizing sacrifice. To better understand how these capacities can be supported and channelled toward the transformations still needed in LTC, the next theme turns to the enduring core of worker dedication and resilience that, while heightened during the pandemic, transcends it.

#### Theme 2: configuring strengths around person-centred care - “Meeting them in those needs”

Building on the elevation of strengths in workers’ immediate collective response to pandemic challenges, this theme configures those same capacities around the stable, core motivational drivers of their commitment to person-centred care. These motivations reflect workers’ personal understandings of quality that guide their best efforts and continue to make LTC work authentically rewarding.

#### Subtheme 2.1: meaningful connections and unique rewards - “it’s really rich work”

Participants illustrated the satisfaction derived from meaningful connections with residents, emphasizing how these relationships uniquely enriched their working lives. Sarah, a recreation therapist, contrasted LTC with her previous experience in less intensive care settings, stating, “Certainly long-term care is where I feel the most intrinsic benefit and gratification.” Ella (clinical nurse leader) described diminished satisfaction in her work life when the pandemic shifted her responsibilities to operational duties and away from direct bedside interactions for several months, and emphasized, “I do this job because I want those connections with the residents and families.”. Comparatively, Sierra, a rehabilitative therapy aide newer to LTC, spoke to the meaning of the ongoing, in-person contact with residents inherent in her role, especially considering the broader societal shift to embracing remote work. Reflecting on her choice, Sierra noted, “I wish I could work from home, but I know I can’t, and I don’t want to either, because I do love my job and I do love helping people.” Sierra illustrated with an anecdote about supporting a resident’s recovery in mobility after several difficult days, and summarized: “to be able to help them achieve their goals is always really satisfying.” Lucy, a spiritual health practitioner, described journeying alongside seniors, especially during end-of-life care, as a privilege. She had not anticipated the extent to which joy and play continue to have enduring importance in residents’ daily lives. She described fulfillment in the following terms:

I love being permitted to hear and to hold the stories, especially around issues of spirituality and meaning, what brings comfort, what brings joy, what people are still trying to come to an ultimate meaning about…it’s really rich work. (Lucy)

Alexa reflected on the fulfilment of getting to know residents in the place they called home, making her work environment one into which she felt honored to be welcomed:

I love working with older adults… they have a lot to offer, mostly on an emotional basis… work becomes a place that feels like another home. (Alexa)

Camila, a spiritual health practitioner, conveyed meanings of reciprocal connection, noting that residents often expressed a desire to “give back too” and were frequently “so thankful and appreciative” even for simple acts like brief one-on-one visits. Accordingly, she challenged assumptions that eldercare work is burdensome or self-sacrificial:

Sometimes I meet people and they find out what I do and they go, ‘Oh, I could never do that. You’re a saint for doing that.’ I actually find it very rewarding. (Camila)

Reflecting further, Camila described a sense of vocational calling: “For many of us… we’re meant to do this work.” Taken together, participants’ accounts highlight connection with residents in their care as a throughline of fulfillment and sustained engagement in LTC work. The emotional reciprocity of caregiving reinforces workers’ personal commitment to high-quality, person-centered care and remains an essential source of resilience.

#### Subtheme 2.2: enacting high quality care - “You’re not going there just to do a job”

Workers spoke of the concrete practices through which they enacted high-quality, dignified, and individualized care. Pandemic-related pressures did not abate their commitment to preserving residents’ dignity and independence or to prioritizing meaningful interactions over task efficiency. These enactments demonstrate how workers translate their intrinsic motivations, personal values, and philosophical beliefs about quality care into everyday actions. Ava, a healthcare support worker, stressed that preserving resident dignity meant going beyond routine care tasks. She described how small gestures, like staying back to color a picture or simply saying good morning, went a long way in allowing residents to maintain pride and dignity:

Like you’re not just going there to do a job, you’re going there to do it with dignity and make sure that they have it themselves. (Ava)

Sophie, an occupational therapy assistant, likewise emphasized the value of supporting residents’ independence through small but meaningful choices, such as deciding how they want their breakfast prepared. For her part, Anna, a music therapist, appreciated her ability to provide individualized care through her music therapy program, especially for residents who struggled to connect or express what they were going through. She believed her personalized approach, which included learning Chinese music and basic language, allowed her to reach residents in meaningful, unique ways:

Being able to meet them in those places, meet them in those needs and just being able to do what I love, helping them out (Anna)

Resident choice and high personal standards for quality care remained central to Lily’s practice as a therapy aide, despite the large number of residents she supported. When large-group programming was suspended during the pandemic, Lily adapted by hosting pop-up recreation events and bringing board games into units. She soon discovered residents often preferred meaningful conversations over activities designed merely to “keep themselves busy.” Lily responded by prioritizing longer, deeper conversations with residents and she resisted her manager’s efficiency-driven expectation that interactions remain under five minutes to maximize the number of residents reached. Sometimes, Lily spent up to a half hour with residents during which there was no ostensible participation in the planned recreation activity:

The big thing for me is recreation is a choice… a lot of the emphasis is on “How many people we see, how many people will come, how much, how much, how much?” But to me, it doesn’t matter… I’m going to stay as long as the person wants to talk to me… the quantity didn’t matter to me, the quality matters. (Lily)

Workers acknowledged that residents entering LTC can feel cynicism and hopelessness, and some depicted their work in terms of transforming residents’ initial perceptions. Chantelle spoke of the joy she felt in helping residents recognize that “life doesn’t come to an end in LTC,” affirming for them that “it’s good that they exist” and that “there are other doors they can open” in this chapter of life. Emilia, social worker, similarly worked to reframe negative perceptions from her distinct vantage point in a transition unit, where residents typically stayed for approximately six months before moving into permanent care. She described residents arriving disillusioned by previous healthcare experiences. As one of their first contacts with facility staff, Emilia strove to demonstrate that LTC workers genuinely cared about their well-being and goals:

They usually come to us quite cynical or tired of the way they’ve been treated. And it’s a great opportunity for me to be able to show them that people in healthcare do care about them, and that we do want them to succeed (Emilia)

Taken together, these accounts show how workers enacted high-quality care through values-driven practice and relationships. Worker efforts to preserve dignity, support independence, and respond to individual needs demonstrate a sustained commitment to principled, personal care. Having elevated workers’ peak strengths in crisis and illuminated the motivations and enactments of person-centred care, the analysis now turns to how these capacities can inform broader systemic improvements through learning, adaptability, and advocacy for change.

#### Theme 3: refractions of strength into learning, growth, and change - “It’s made Us stronger”

This theme is an examination of how the strengths elevated in crisis (Theme 1) and configured around person-centred care (Theme 2) refract outward into forward-facing orientations. It begins with a consideration of how workers identified opportunities to improve their workplaces and adapt LTC as an evolving, learning system. It then profiles how these capacities surfaced through workers’ advocacy through which workers articulated concerns and aspirations for improving LTC for both residents and staff.

#### Subtheme 3.1: the learning in adaptation - “I like that part, that there’s always learning”

As the pandemic progressed, workers embraced evolving workplace needs as opportunities to rethink established practices and contribute to improving care environments. Naomi, a social worker, found herself taking on a more hands-on role implementing infection control measures and redesigning public space layouts. She reflected positively on the continuous learning involved in navigating this unfamiliar territory:

This is all new to everyone, and no one really knows where we’re going. So we are all learning and I like that part. (Naomi)

Other workers described similar engagement with learning opportunities, recognizing moments for growth within the distinct role changes necessitated by the pandemic. Kayla, a COVID-19 support lead, described the pandemic inducing a shift in her longstanding position as an adult day program manager to a role where she worked more directly with staff rather than clients and family caregivers, calling this transition both a “nice change of scenery” and “a really good learning opportunity.” Recalling Ella’s (clinical nurse leader) earlier account of diminished satisfaction when her clinical role shifted to of operational coordination, she described pragmatically adapting “from a more collaborative style of leadership to a more directive style” during outbreaks. Reflecting on this period of her life, she found she learned “a lot about what [she’s] capable of” and what strategies proved effective.

Cynthia, occupational therapist, saw her role expand into new responsibilities for infection control, housekeeping oversight, and facility-wide safety coordination. Reflecting positively on the reshaped parameters of her job, Cynthia noted gaining a “bigger picture” perspective of how a LTC facility runs, which improved her capacity for holistic decision-making. At the same time, she pointed to these expanded responsibilities as significantly heightening her stress levels and limiting her ability to adequately provide staff safety education, something she viewed as critically important.

Other workers similarly described the positive value of embracing new learning, while frankly acknowledging its trade-offs, particularly the unsustainability of this learning in context of ongoing workload strain. Cate described a journey of adapting to eight managerial changes during the pandemic, repeatedly reorienting as the “middle person” between shifting management styles and staff needs. Tyler, a Director of Care, described how successive COVID-19 outbreaks compelled him to rapidly build expertise in infection control, contact tracing, and resident cohorting. While he acknowledged becoming “more intelligent” about managing outbreaks, this knowledge came at the expense of working over 100 extra hours in three months, leaving him reflective and struggling to find any other positives:

I didn’t have much left in the tank, so to speak…I’m having a tough time finding any [positive changes]. Other than knowledge gained. (Tyler)

Tyler’s account, along with those of others described here, provokes recognition that some forms of learning in the navigation of pandemic-driven uncertainty were experienced as more depleting than transformative. Nonetheless, the shared capacity to adapt reflects activation of latent strength in workers’ willingness to learn and rethink practices in a novel and unasked-for conditions. Given how much of this learning was highly stressful and driven by necessity rather than choice, the persistence workers demonstrated and the framing of this strength in positive terms are striking.

#### Subtheme 3.2: advocating change from within – “we’re going to have to do a lot of work”

Workers’ strengths refracted outward in advocacy perspectives informed by firsthand knowledge of the structural issues intensified and made newly visible by the pandemic. In their candid accounts of the toll exacted by extraordinary commitments to care, workers consistently identified chronic staff shortages as a key area for reform, both in terms of workforce sustainability and future crisis preparedness. In response to these strains, Emilia called for clearer standards around role definition, workload monitoring, and organizational accountability to prevent worker dedication from being mistaken for unlimited capacity. As she put it, there was “no one around to say you’re taking on too much”.

The need to better protect workers’ innate commitment to care extends beyond workload concerns to the quality of care itself. Drawing on extensive career experience, Rhonda reflected on the ideals of person-centered care in LTC, and how, in her view, certain elements of the public health pandemic response “totally negated” the aspirations of those ideals. She pointed to the social confinement of residents as one example, describing its traumatizing, deconditioning impact on residents and how implementing such protocols was “very, very difficult for staff.” Rhonda framed the pandemic as renewing an imperative for culture change and a holistic approach to quality of life. She further specified this in terms of listening to worker experiences and supporting workers, summarizing: “we’re going to have to do a lot of work in that area”. Rhonda’s broad account of what workers endured aligns with the direct experience of Shannon, a young registered nurse hired at the onset of COVID-19, whose entry into nursing was colored entirely by the constraints of pandemic-era care. Shannon highlighted the stakes of person-centered care clashing with restrictive protocols by describing an outbreak in which visitors were prohibited, including for residents receiving end-of-life care. Although digital visits and calls were offered, it was common for these residents to lack the dexterity or cognitive ability to connect with family members through these means. Reflecting on the distress created by this situation, Shannon said she deeply empathized with families, imagining if it were her own grandfather, her “automatic instinct would be to get them out of the facility.” Then, placing herself in the resident’s position, she stated, “I would rather die with people that I love than to die out of fear, and to die alone.” Shannon’s advocacy for more compassionate, person-centered conditions around visitation points to a broader societal fault line of polarization of beliefs about visitation during the pandemic. It also conveys the emotional and ethical weight of navigating that divide. Without passing a value judgment on the rightness of Shannon’s view, her advocacy reflects the strength of workers’ personal standards and the sincerity with which care can be given.

Other workers also voiced a desire for change as they navigated tensions within the structural and operational realities of LTC. Naomi critically reflected on the enforcement-oriented nature of her role as a liaison between management, healthcare policy, and residents’ families, describing feeling “torn” between being an “agent of the system” and an “agent of change”. For her, “change” meant advancing compassionate flexibility and responsiveness within the countervailing realities of LTC, imposed in this case by the public health measures. In her role as a health care aide, Driya quietly defied her workplace’s recommendation to suspend resident showers during an outbreak. Although “everybody took it as the rule” and many colleagues seemed relieved to have one less task to do, Driya was troubled by what she saw as an unacceptable compromise to care quality and continued offering showers whenever time and energy allowed. Her dissent illustrates advocacy in the form of unsung frontline acts that preserved care standards within ambiguous ethical conditions.

Minsheng, a Clinical Operations Supervisor, offered a forward-looking critique and vision exemplifying how workers’ synthesized strengths can inform broader systemic change. Reflecting on what the pandemic taught him about the LTC sector’s place in society, he observed, “we’re connected much deeper than what I thought we were”. Minsheng described the pandemic as a long-needed “wake up call” to that reality. He noted that most members of the public had previously been unaware of “the inside stories” of LTC, but the pandemic had “exposed [stories] to the level that we never imagined”, revealing status quo conditions as “far, far below the general public’s expectations.” Pointing to the exposed structural deficiencies in LTC, specifically funding limitations, staffing models and ratios, and weaknesses in coordination and information-sharing, Minsheng saw heightened public awareness of these as critical to repositioning LTC as a “competing priority” alongside acute care and other healthcare sectors. He emphasized the need for government action as essential to bringing about the change he envisions:

I wanted to see some changes positively from our government level and to fund our long-term care… to the deepest and longest level. (Minsheng)

In calling for a broader societal and political awakening, Minsheng framed the public imaginary of the sector as essential to realizing and amplifying LTC worker strengths more widely. Collectively, the perspectives of workers articulate a vision of their strengths, first elevated through crisis responses, then configured around person-centred care commitments, and finally refracted outward in capacities for innovation and system-level advocacy. In these ways, members of this workforce have themselves illuminated pathways towards its strong future.

## Discussion

Canada’s LTC sector faces a post-pandemic reckoning with workforce sustainability, worker wellbeing, and the growth needed to meet projected demand. While these challenges merit continued scholarly and policy attention, critical voices have noted a saturation of research focused on identifying and cataloguing the problems, and making the recommendations, that are by now familiar [11]. In dialogue with this concern, our research drew on Appreciative Inquiry concepts of elevation, configuration, and refraction to present a strengths-based reframing of LTC workers’ experiences during a period well documented as one of peak challenge and strain for the sector [21]. In practical terms, this reframing interpreted worker strengths as a cohesively expressed, renewable system asset that stabilizes care delivery, enacts person-centred quality, and generates implementable guidance for service and policy redesign when appropriately recognized and resourced.

Appreciative Inquiry offered a distinct perspective on worker strengths. However, the strengths we identified align with established knowledge about LTC worker resilience, purpose, and dedication. Czuba et al. [29] found that aged care support workers maintained strong emotional connections to their caregiving roles, routinely exceeding the boundaries of their formal job descriptions even in the face of ongoing stress and a persistent sense that their roles and expertise were undervalued by management and clinical colleagues. Workers’ prioritization of emotional connections was prominent in our previous analysis of this dataset, which examined mental health support needs and found that peer debriefs and group discussions among workers, while not universally welcomed, were regarded by many as critical sources of emotional and relational support during the pandemic [3]. This support was most effective when spaces were voluntary, emotionally safe, and embedded in the rhythms of daily workplace routines. Similarly, authors of a large cross-sectional study of over 3,700 care aides in Western Canada found that psychological empowerment was positively associated with informal peer interactions, such as spontaneous information-sharing and emotional support, at the same time that formal interactions were negatively associated with care aides’ sense of work meaning [34]. Our findings about workers’ capacities for adaptability and reflective practice extended beyond crisis response into improvisation, systems-level thinking, and learning. These qualities resonate with prior qualitative findings showing that aged care staff across diverse roles demonstrate leadership characterized by curiosity, improvisation, and real-time decision-making in resident care, challenging views of LTC work as regimented, task-oriented, and unimaginative [45]. These strengths, as in our analysis, reflected a deliberate drive to improve care quality and influence change from within the system. Our study thus adds a strengths-based voice to the pandemic LTC literature while extending the broader literature on LTC worker strengths through an articulation of these capacities as a cohesive and enduring system asset whose full expression can renew workforce well-being and drive sector transformation.

### Implications

We add our voices to those of researchers calling on governments to increase and standardize the number of hours of direct care provided per resident per day in LTC. Knowing that higher staffing levels improve quality of care, the Health Standards Organization recommends a minimum 4.1 h [46] and the Royal Society of Canada calls for 4.5 h [11]. Some experts suggest that up to 6 h may be necessary for high-quality care [47]. Though some Canadian provinces such as Ontario and Nova Scotia have publicly pledged to reach at least 4 h [48, 49], this benchmark remains unmet nationwide as of this writing. In provinces where most of our participants lived, the latest publicly reported averages are in the mid-three-hour range, for example, 3.43 h in B.C [50]. and 3.62 funded worked hours in Alberta [51]. Additionally, Alberta’s 2024 legislation for funded hours has removed the requirement for a measurable numeric minimum of direct care hours [52].

Reynolds [53] cautions that while recognizing workers’ strengths is important, such admiration must not take the place of structural accountability or the collective ethical obligation for needed transformations of the conditions under which care is provided. As such, the case for increasing funded hours of direct care is especially important in the context of our study. Reynolds also resists pathologizing burnout as a failure of personal resilience and instead frames it as a signal of injustice. Accordingly, for all our focus on worker strengths, we press the point that the onus is not on workers to overcome the structural, upstream drivers of workplace problems. Rather, this ethical obligation lies with governments, policymakers, and organizations to resource healthy work environments so that workers may receive the support and resources necessary to flourish. Regarding this flourishing, we hypothesize that funding more direct care time will lead to improvements not fully captured by staffing benchmarks, for two reasons. First, a key inflection point sustaining the “crisis ordinary” workforce holding pattern appears to be burnout directly attributed to chronic short-staffing, which in turn is a major driver of workforce attrition [19]. A funding scenario that decisively ameliorates this source of burnout would clearly reduce attrition and improve care quality. Beyond this, it could also act as a tipping point for a reinforcing cycle of improvement by further unlocking workers’ latent strengths for organizational and system-level change. Our findings on advocacy perspectives showed that, when supported and channelled appropriately, this capacity extends beyond individual flourishing and can contribute to broader transformation, including changes that help attract and retain more workers.

Second, increased funding would implicitly convey recognition of workers’ value, given the significant investment required to achieve even four hours of direct care per day. This recognition is particularly important given how LTC work can be rendered invisible through structural misrecognition, understood here as system-level undervaluing that obscures workers’ social and professional contributions [36]. As our findings showed, this invisibility may stem in part from workers independently identifying and addressing problems as they arise. It also reflects the nature of LTC work itself, which typically occurs behind closed doors in one-on-one interactions with residents who, due to cognitive impairments such as dementia, may be unable to recognize or respond to worker contributions [54]. Beyond residents, recognition and its absence reach workers through multiple channels. These include workplace relationship dynamics with colleagues, supervisors, and leaders [55], as well as sources external to the workplace such as close personal relationships, members of the lay public, and cultural narratives, including media portrayals that influence public interpretations of workers’ roles [56]. All these affect how workers perceive their value and, in turn, influence motivation to remain in the sector. The pandemic generated evidence of public and institutional appreciation temporarily bolstering healthcare workers’ well-being, yet this recognition and its benefits proved short-lived when not anchored in structural change [32]. To carry this lesson forwards, we call for greater resourcing of workers combined with persuasive messaging and imaginative storytelling that overwrites the shallow “saintly but low-skilled” cultural narrative trope, which both romanticizes and diminishes LTC work. Supplanting this narrative with one that recognizes LTC workers as skilled professionals who deserve honour, support, and adequate resources can seed workers’ experiences of recognition in multiple ways, reinforcing self-confidence, self-respect, and self-esteem [56]. These improvements can enhance care quality and workforce stability in ways that exceed what might be expected from material investment alone.

## Limitations

This study has limitations. First, it is a secondary qualitative analysis in which Appreciative Inquiry was applied retrospectively to interview data originally generated to examine worker mental health, moral injury, and pandemic preparedness rather than to explicitly elicit strengths-based accounts. As a result, worker strengths were not consistently articulated as such within participants’ accounts and were at times embedded implicitly in narratives oriented toward stress, constraint, and preparedness. Although Appreciative Inquiry does not require prospective use to be analytically valid, its retrospective application here entails the likelihood that additional dimensions of worker strength would have been identified under a study design explicitly oriented toward strengths from the outset. Moreover, the secondary analysis limited the iterative and co-constructive capacities for which Appreciative Inquiry research designs are known and that depend on ongoing engagement with participants. Second, voluntary recruitment during the pandemic may have constrained participation among workers most depleted by sustained crisis conditions. Accordingly, these findings should not be taken as representing the full LTC workforce or the full range of worker strengths but instead offer an empirically anchored and theoretically informed foundation for future research that prospectively integrates Appreciative Inquiry into study design, leadership development, and policy-relevant knowledge production.

## Conclusions

Taken together, this study offers a strengths-focused theoretical contribution that enriches critical scholarship in LTC and related frontline care work centred on positive concepts like joy [38] and justice [53]. It also adds complementary value to postpositivist traditions in health services research that examine measurable constructs such as LTC worker empowerment, resilience, job efficacy, and related dimensions [26, 27, 34].

Looking ahead, this theoretical orientation has clear implications for how knowledge is generated, mobilized, and governed in the LTC sector. Integrating this perspective into primary research designs, leadership development, and policy processes can help build care environments that advance worker well-being and person-centred care as intrinsically linked rather than competing aims. These efforts may also help embed LTC worker perspectives into research infrastructure, which could include workers’ involvement in policy committees, research agenda-setting, and co-facilitating community–academic partnerships. Scaled nationally, such infrastructure could also help address the sector’s persistent lack of high-quality, pan-Canadian data on LTC workers’ quality of work life, including mental health and wellbeing, a foundational gap identified in the *Restoring Trust* briefing [31]. While that recommendation prioritized measurable indicators, qualitative social science grounded in worker perspectives and informed by theories of positive change such as Appreciative Inquiry can make a complementary contribution. In sum, policy must act to stop depleting workers’ strengths, and research agendas should center workers’ knowledge and aspirations to revitalize the evidence base guiding transformation in the sector.

## Supplementary Information

Below is the link to the electronic supplementary material.

Supplementary Material 1

## Data Availability

The qualitative datasets generated and/or analysed during the current study are not publicly available due to the potential for participant identification, but de-identified excerpts supporting the analysis are available from the corresponding author on reasonable request.

## Abbreviations

LTC: Long-Term Care
