# Supplementary material for: “You’re not just there to do a job”: a qualitative examination of Canadian long-term care worker strengths
Source: BMC Health Serv Res. 2026 Feb 25;26:435. doi: 10.1186/s12913-026-14197-8 (PMC13041297; doi:10.1186/s12913-026-14197-8)
Supplement: Supplementary file 1 — Supplementary Material 1 [file 12913_2026_14197_MOESM1_ESM.pdf]

Additional file 1

Interview Guide

Socio Demographic:

- What is your name and age?
- What is the name of your organization and what is your current role?
- How many years have you been working in this field?
- Are you a full-time, part-time, casual, or a “pandemic hire” employee?
- What is your marital status?
- How many dependents do you have living in your home? Are they children? Elders? Other?
- What is your highest level of education?

Understanding of Role and Role Changes during the Pandemic:

- Please describe your current role in your organization.
- What is satisfying or positive about your job? What were stressors for you at work before the pandemic began?
- How would you describe your level of understanding about the pandemic? I.e. how the virus is spread, the need for PPE, how to wear PPE; was there enough PPE; how was PPE provided? How were new rules and protocols communicated?
- How has your role changed since the pandemic? Prompt: Positive changes? Negative changes?
- Please describe difficulties you faced during the pandemic.
- What have you found to be the most upsetting aspect of working in a pandemic?
- How has the pandemic impacted your interactions with residents and family members? With your co-workers and supervisors?
- Has working during the pandemic left you feeling guilty in any way? If so, could you please explain?
- Overall, how has the pandemic impacted your ability to fulfill your role in your job?
- Have you had any vacations cancelled because of being on outbreak?
- What is the longest consecutive amount of time you’ve worked during the pandemic?

Managing Stress:

- What resources are you using to stay well?
- What helps you manage difficulties: Prompt: within your organization, what resources and benefits have been provided?
- What resources do you wish you had?
- Who has supported you within the workplace?

Personal Impacts:

- How has the pandemic affected your life outside of work?
- (Holding in view dependents noted during earlier question) Who do you live with, what sort of role do you play in your family?
- How has the pandemic impacted your stress levels and responses outside of work?
- Who supports you outside the workplace?

Recommendations:

- Can you provide us with recommendations for how to be better prepared for another global pandemic?
- Can you explain the process of obtaining your vaccine?
- Is there anything else that you feel is important to share with us?
